# ProbPS: A new model for peak selection based on quantifying the dependence of the existence of derivative peaks on primary ion intensity

**DOI:** 10.1186/1471-2105-12-346

**Published:** 2011-08-17

**Authors:** Shenghui Zhang, Yaojun Wang, Dongbo Bu, Hong Zhang, Shiwei Sun

**Affiliations:** 1 Institute of Computing Technology, Chinese Academy of Sciences, Beijing, 100190, China; 2 Zhejiang Gongshang University, Zhejiang, 310018, China

## Abstract

**Background:**

The analysis of mass spectra suggests that the existence of derivative peaks is strongly dependent on the intensity of the primary peaks. Peak selection from tandem mass spectrum is used to filter out noise and contaminant peaks. It is widely accepted that a valid primary peak tends to have high intensity and is accompanied by derivative peaks, including isotopic peaks, neutral loss peaks, and complementary peaks. Existing models for peak selection ignore the dependence between the existence of the derivative peaks and the intensity of the primary peaks. Simple models for peak selection assume that these two attributes are independent; however, this assumption is contrary to real data and prone to error.

**Results:**

In this paper, we present a statistical model to quantitatively measure the dependence of the derivative peak's existence on the primary peak's intensity. Here, we propose a statistical model, named ProbPS, to capture the dependence in a quantitative manner and describe a statistical model for peak selection. Our results show that the quantitative understanding can successfully guide the peak selection process. By comparing ProbPS with AuDeNS we demonstrate the advantages of our method in both filtering out noise peaks and in improving *de novo *identification. In addition, we present a tag identification approach based on our peak selection method. Our results, using a test data set, suggest that our tag identification method (876 correct tags in 1000 spectra) outperforms PepNovoTag (790 correct tags in 1000 spectra).

**Conclusions:**

We have shown that ProbPS improves the accuracy of peak selection which further enhances the performance of de novo sequencing and tag identification. Thus, our model saves valuable computation time and improving the accuracy of the results.

## 1 Background

Mass spectrometry is a popular method for protein identification [1-6]. In a typical protein identification experiment using mass spectrometry, proteins are first digested into peptides by an enzyme, say *trypsin*. Tandem mass spectra of the peptides are generated using a tandem mass spectrometer (MS/MS). Traditionally, two approaches for peptide identification from MS/MS spectra have been used: database searches [3-8] and *de novo *sequencing [9-31].

Typical database searches first identify a set of candidate peptides from a protein sequence database, and then construct a theoretical spectrum for each peptide. Finally, the similarity between the theoretical spectrum and the MS/MS experimental spectrum is calculated and the most similar peptides are reported as predictions. There are several popular tandem mass spectrometry data analysis programs of this type: SEQUEST [3], Mascot [4], X!Tandem [5], SCOPE [6], and ProbID [7], are some examples of these. Before comparing a theoretical spectrum against an experimental spectrum, noise peaks in the experimental spectrum should be filtered out. Noise peaks in the spectrum can cause significant differences between the experimental and theoretical spectra and, as a result, correct solutions may be missed.

*De novo *sequencing, on the other hand, is database-independent because it exclusively uses the information contained in the MS/MS spectrum. Thus, the *de novo *technique has the potential to identify peptides that are not included in protein sequence databases. Widely-used *de novo *packages include PEAKS [9,10], PepNovo [11,12], et al. [13-31] Recently, variants of *de novo *sequencing, the tag-based methods [32-38], have been developed to identify a segment of a peptide rather than a full-length peptide. After inferring the tags from a MS/MS spectrum, the candidate peptides that do not match any of the tags are filtered out. Therefore, an effective tag identification method can improve identification accuracy and reduce the running time for database searches by reducing the number of candidate peptides. Both *de novo *methods and tag-based methods usually require high-quality spectra, and do not perform well on spectra with noise peaks. Thus, peak selection is important for the effective use of *de novo *methods.

Generally speaking, there are three types of peaks in a tandem mass spectrum: i) the primary peak that is highly likely to be accompanied by a set of derivative peaks caused by the loss of ammonia, the loss of water, or isotopic shift; ii) noise peaks from signals from mass spectrometry and other unknown reasons; and iii) peaks generated from contaminants. Although isotopic shifts and neutral losses are often observed for peaks generated from contaminants, complementary peaks are seldom observed. This provides a way to distinguish valid peaks from noise and contaminant peaks. In this study, the latter two peaks are called noise peaks.

Before attempting to identify a peptide from a MS/MS spectrum, it is useful to perform a pre-processing step (called peak selection) to filter out noise and contaminant peaks. A widely accepted peak selection rule utilizes two peak attributes, peak intensity and the existence of derivative peaks. Briefly, a peak accompanied by derivative peaks and an associated complementary peak is likely to be valid; peaks without these features are likely to be noise. Our observations suggest that the existence of derivative peaks and complementary peaks is strongly depending on the primary peak intensity. Existing methods for peak selection adopt simple models that assume that these two attributes are independent. This assumption contradicts to real data and is error prone. In this study we proposed a statistical model, named ProbPS, to capture the interdependence of peak intensity and the existence of derivative peaks in a quantitative manner. Our experimental results demonstrate that our model can improve both peak selection and tag identification.

## 2 Methods

### 2.1 Notation

For a peak *p *in a tandem mass spectrum,

• *V *= 1 if the peak is a valid primary peak; otherwise *V *= 0.

• *I *is the peak intensity;

• *ISO *indicates the existence of isotopic shift;

• *NH*_3 _indicates the existence of a peak that corresponds to the neutral loss of an ammonia;

• *H*_2_*O *indicates the existence of a peak that corresponds to the neutral loss of a water;

• *COMP *indicates the existence of a peak that corresponds to a complementary ion;

### 2.2 The model for peak selection

#### 2.2.1 Quantifying the dependency of derivative ions on primary peak intensity

To investigate the dependency of derivative ions on primary peak intensity we used spectra from the Swed-CAD database [39], a collection of high quality MS/MS spectra of tryptic peptides. Using SEQUEST, we identified 15,897 unique, annotated peptide-spectrum matches (PSM) to use as a training set.

**Figure 1:**
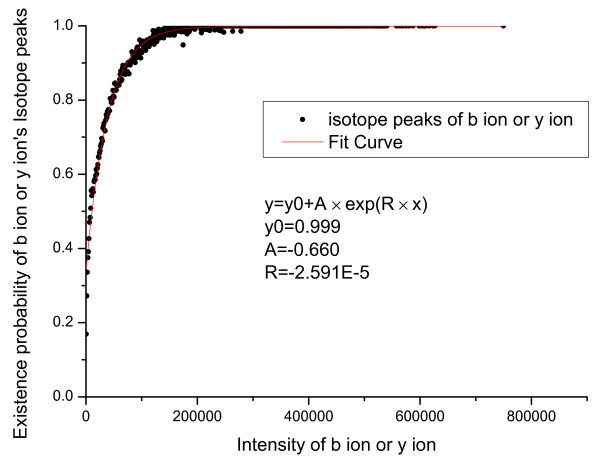
**Emperical density function of *P*(*ISO*|*I*, *V *= 1)**. Here, *P*(*ISO*|*I*, *V *= 1) is approximated by an exponential function *y *= *y*_0 _+ *A × exp*(*R × x*).

**Figure 2:**
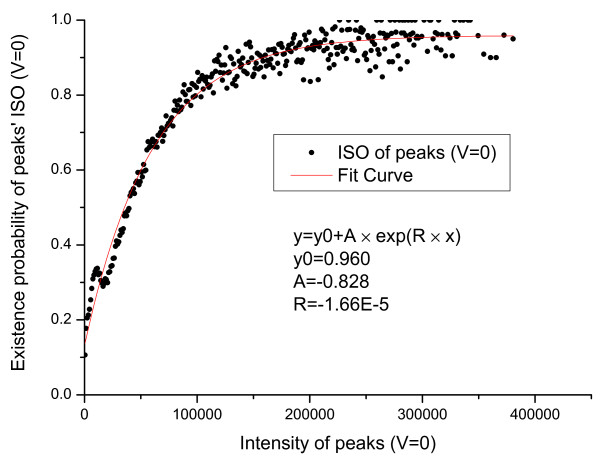
**Emperical density function of *P*(*ISO*|*I*, *V *= 0)**. Here, *P*(*ISO*|*I*, *V *= 0) is approximated by an exponential function *y *= *y*_0 _+ *A × exp*(*R × x*).

**Figure 3:**
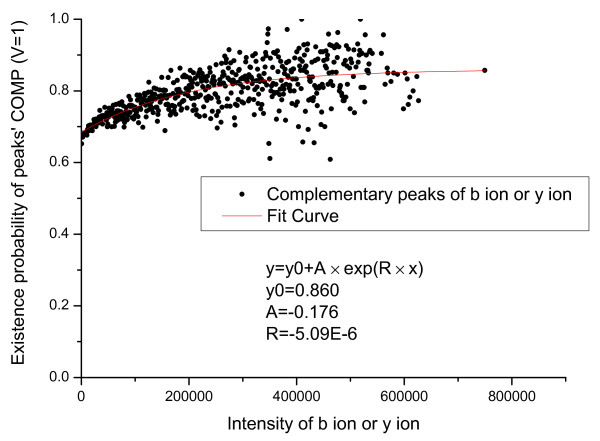
**Emperical density function of *P*(*COMP*|*I*, *V *= 1)**. Here, *P*(*COMP*|*I*, *V *= 1) is approximated by an exponential function *y *= *y*_0 _+ *A × exp*(*R × x*).

**Figure 4:**
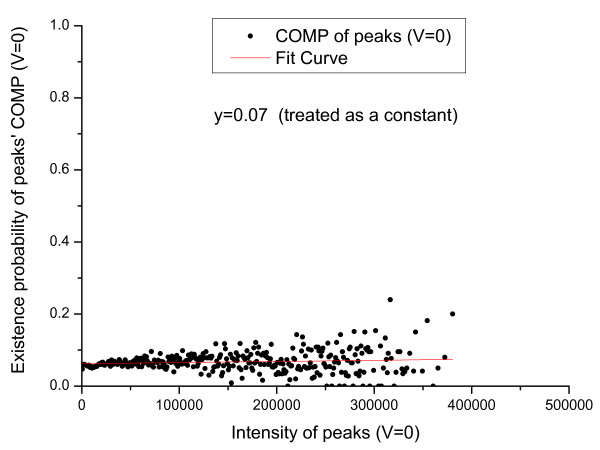
**Emperical density function of *P*(*COMP*|*I*, *V *= 0)**. Here, *P*(*COMP*|*I*, *V *= 0) is approximated by a constant.

We first count the number of valid primary peaks with an intensity *I *(*N_total_*(*I*)) in the training set. From the valid primary peaks, the peaks having isotopic shift were identified and counted (*N_ISO_*(*I*)). The probability that a valid primary peak has an isotopic shift can then be estimated as $P\left(ISO|I,V=\text{1}\right)=\frac{N_{ISO}\left(I\right)}{N_{total}\left(I\right)}$. Similarly, *P*(*ISO*|*I*, *V *= 0), *P*(*COMP*|*I*, *V *= 1) and *P*(*COMP*|*I*, *V *= 0) were estimated and the results are shown in Figure 1, 2, 3, 4.

In Figure 1 an evident nonlinear relationship between primary peak intensity and the existence of isotopic peaks can be observed. The nonlinear relationship can be explained by supposing that, for a primary ion, its isotopic derivative is observed with probability *p*. Then, for a total of *I *primary ions, an isotopic derivative would be observed with probability $1-{\left(1-p\right)}^{I}=1-e^{\frac{I}{p}}$. Therefore, it is reasonable to approximate this relationship using an exponential function. Like *P*(*ISO*|*I*, *V *= 1), *P*(*ISO*|*I*, *V *= 0) also approximates 1 as the peak intensity goes to infinity. The reason for the slight differences in Figure 1 and 2 is that a contaminant ion might generate an isotopic shift similar to the shift generated by a primary ion. A significantly different pattern between *P*(*COMP*|*I*, *V *= 1) and *P*(*COMP*|*I*, *V *= 0) is observed (Figure 3 and 4) because for contaminant ions, complementary peaks are seldom generated.

**Figure 5:**
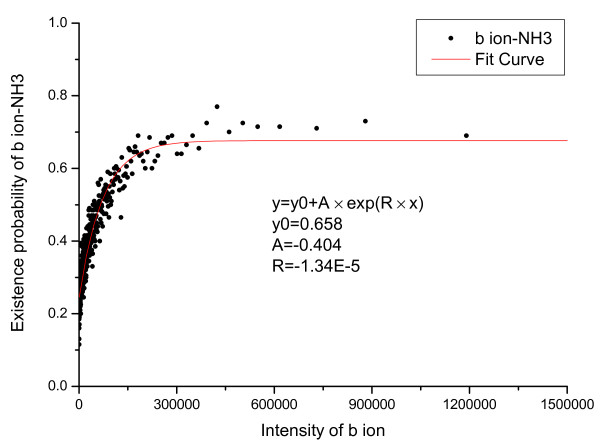
**Emperical density function of *P*(*NH*_3_|*I*, *V *= 1) for *b *ions**.

**Figure 6:**
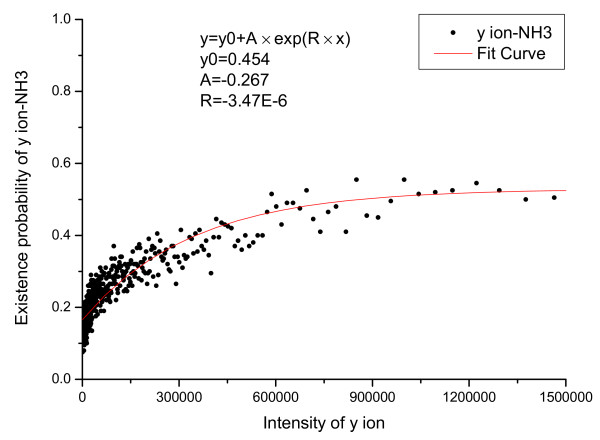
**Emperical density function of *P*(*NH*_3_|*I*, *V *= 1) for *y *ions**.

**Figure 7:**
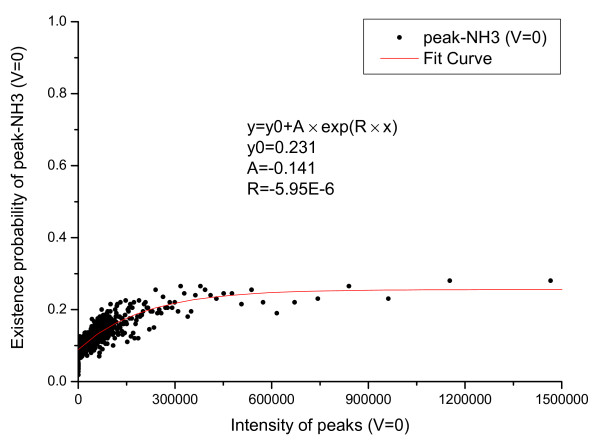
**Emperical density function of *P*(*NH*_3_|*I*, *V *= 0)**.

**Figure 8:**
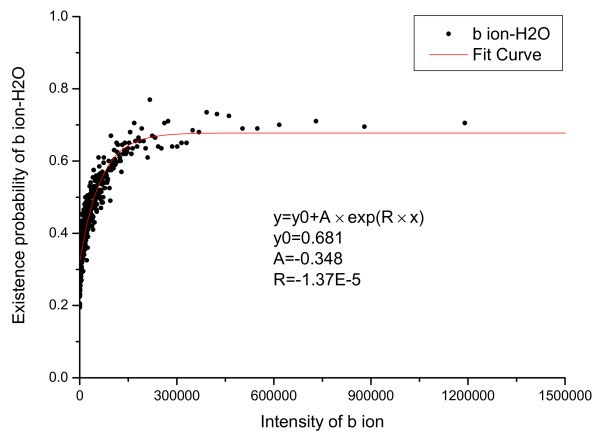
**Emperical density function of *P*(*H*_2_*O*|*I*, *V *= 1) for *b *ions**.

**Figure 9:**
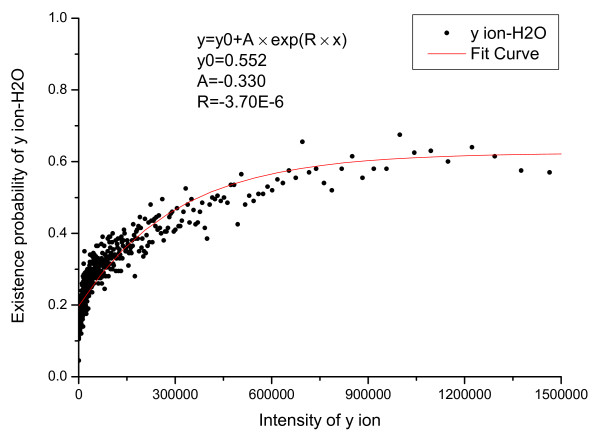
**Emperical density function of *P*(*H*_2_*O*|*I*, *V *= 1) for *y *ions**.

**Figure 10:**
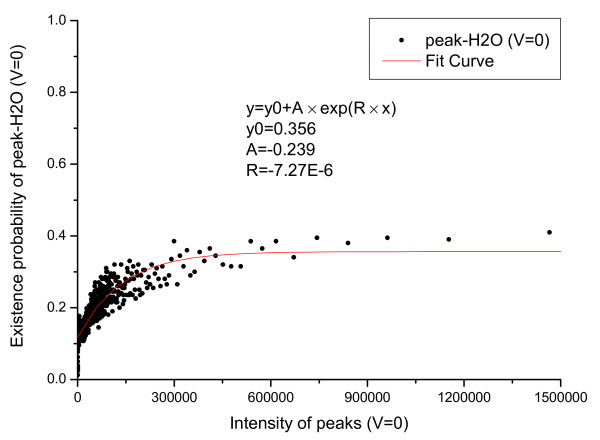
**Emperical density function of *P*(*H*_2_*O*|*I*, *V *= 0)**. All functions are approximated by an exponential function *y *= *y*_0 _+ *A × exp*(*R × x*) and the parameters of the functions are listed in the figures.

The relationship between derivative peaks related to neutral losses and primary peak intensity were also calculated and are shown in Figure 5, 6, 7, 8, 9, 10, where *b*-ion and *y*-ion are listed separately because they differ in the possibility of neutral losses. The results in the figures indicate that *P*(*ISO*|*I*, *V *= 1) approximate 1 as the primary peak intensity goes to infinity. On the other hand, *P*(*NH*_3_|*I*, *B*) and *P *(*NH*_3_|*I*, *Y *) approximate a number smaller than 1 and so do *P*(*H*_2_*O*|*I*, *B*) and *P *(*H*_2_*O*|*I*, *Y *). The reason for this is that neutral losses are related to the composition of the amino acid ions. Some amino acids can lose ammonia or water, while others cannot [8]. In our study, we have introduced a scale factor to capture the influence of the amino acid composition on neutral losses. Figure 5, 6, 8, and 9 support the earlier observation that *b*-ions are more likely to have neutral loss than *y*-ions [40,41]. In summary, noise peaks usually show different patterns from valid peaks, and this observation presents an opportunity for valid peak selection.

#### 2.2.2 Bayesian framework for peak selection

A quantitative description of the dependences was employed to develop a Bayesian framework for peak selection. Let *P*(*V *= 1|*I*, *D*) denote the probability that a peak is valid given two types of peak attributes, peak intensity *I*, and the existence of derivative peaks *D *= {*ISO*, *NH*_3_, *H*_2_*O*, *COMP *}. Then, *P *(*V *= 1|*I*, *D*) was estimated as follows:

$$\begin{array}{c} \;\;P\left(V=1|I,D\right)\\ =\frac{P\left(I,D|V=1\right)P\left(V=1\right)}{P\left(D\right)}\\ =\frac{p^{\left(V=1\right)}}{p^{\left(V=1\right)}+p^{\left(V=0\right)}} \end{array}$$

where *p*^(*V *= 1) ^= *P*(*I*, *D*|*V *= 1)*P*(*V *= 1) and *p*^(*V *= 0) ^= *P*(*I*, *D*|*V *= 0)*P*(*V *= 0).

Since derivative peaks are variants of primary ions, it is reasonable to assume the independence of different derivative peaks. Therefore, the numerator of the above fraction can be decomposed as:

(1) $$\begin{array}{lll} p^{\left(V=1\right)} & =P\left(I,D|V=1\right)P\left(V=1\right)\; & \text{(1)}\\ & =P\left(I|V=1\right)P\left(V=1\right)\underset{d\in D}{\prod }P\left(d|I,V=1\right)\; & \text{(2)}\\ & =P\left(V=1|I\right)P\left(I\right)\underset{d\in D}{\prod }P\left(d|I,V=1\right)\; & \text{(3)}\\ & \; & \text{(4)} \end{array}$$

Similarly, the denominator can be rewritten as:

(2) $$\begin{array}{lll} p^{\left(V=1\right)} & =P\left(I,D|V=0\right)P\left(V=0\right)\; & \text{(1)}\\ & =P\left(I|V=0\right)P\left(V=0\right)\underset{d\in D}{\prod }P\left(d|I,V=0\right)\; & \text{(2)}\\ & =P\left(V=0|I\right)P\left(I\right)\underset{d\in D}{\prod }P\left(d|I,V=0\right)\; & \text{(3)}\\ & \; & \text{(4)} \end{array}$$

Finally, the following approximations were obtained:

$$\begin{array}{c} \;P\text{(}V=1|I\text{, }D\text{)}\\ =\;\frac{\prod_{d\in D}P\left(d|I,V=1\right)}{\prod_{d\in D}P\left(d|I,V=1\right)+r_{v}\times \prod_{d\in D}P\left(d|I,V=0\right)} \end{array}$$

where *r_v _*= *P *(*V *= 0|*I*)*/P *(*V *= 1|*I*).

$$\begin{array}{c} \;P\text{(}V\text{ = 0}|I\text{, }D\text{)}\\ =\;\frac{\prod_{d\in D}P\left(d|I,V=0\right)}{\prod_{d\in D}P\left(d|I,V=0\right)+r_{v}\times \prod_{d\in D}P\left(d|I,V=1\right)} \end{array}$$

Where $r_{n}=P\left(V=1|I\right)∕P\left(V=0|I\right)=\frac{1}{r_{v}}$.

**Figure 11:**
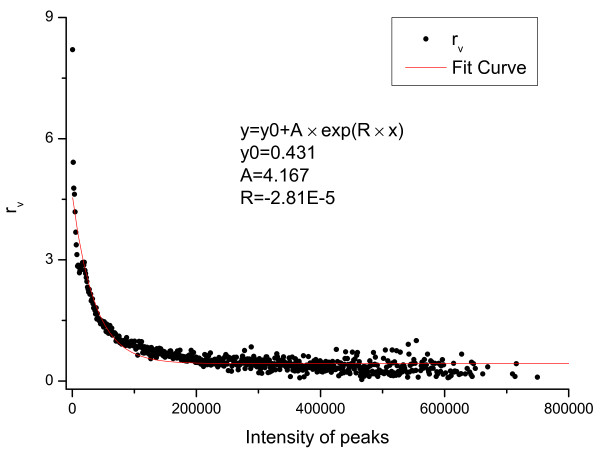
**Distribution of *r_v_*(*I*) calculated using the data set from the **SwedCAD**database**.

The relationship between *r_v_*(*I*) and *I *was calculated using the data set obtained from SwedCAD and the results are shown in Figure 11. A clear geometric distribution was obtained. Similar results were also obtained using data sets from Keller's lab [42] (See Additional File 1: figure S1).

## 3 Results

### 3.1 Peak selection based on *probPS*

**Figure 12:**
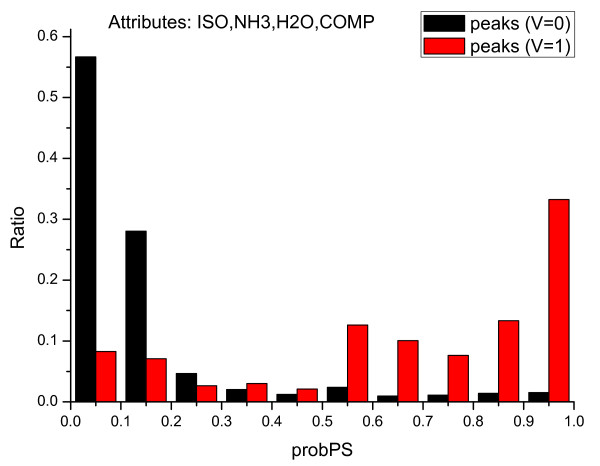
**Distribution of *P*(*V *|*I*, *ISO*, *NH*_3_, *H*_2_*O*, *COMP *) acquired from **SwedCAD**data set**. From this plot, it is obvious that a peak with *probPS *≥ 0.5 tends to be valid.

We use *P*(*V *= 1|*I*, *D*) (denoted as *probPS*) to determine whether or not a peak was valid. For each peak in the training spectra, *probPS *was calculated, and the distribution of *probPS *is summarized in Figure 12. It can be observed that a valid primary peak usually has a high *probPS *value, while a noise peak usually has a low *probPS *value. Further, peaks with *probPS *≥ 0.5 are highly likely to be valid. Therefore, we can utilize the posterior probability to distinguish valid peaks from noise ones. For instance, peaks with higher *probPS *can be selected to execute the *denovo *algorithm or for tag identification. The *probPS *score can also be used to improve database searches by filtering out invalid peaks.

We also compared *probPS *against the *relevance *value used in AuDeNS [43]. AuDeNS uses a framework for *de novo *sequencing of peptides. It first cleans the input spectrum with a number of data cleaning algorithms ("grass mowers"), followed by a sequencing algorithm. It applies the mowers to the input data, assigning to each input peak *i *a *relevance *value *r*(*i*), with the default being *r*(*i*) = 1. Hereby, each mower *M *uses a *relevance *factor *Rel_M _*(which can be set as a parameter of AuDeNS), and the *relevance *value of peak *i *is then given by $r\left(i\right)=1+\sum_{M\;\in \;mowers}Rel_{M}\cdot M\left(i\right)$, where *M *(*i*) is the value assigned to peak *i *by mower *M*. The *relevance *of a solution is then the sum of the relevances of the peaks matched by this solution. Precisely, AuDeNS produces a ranked list of sequence suggestions for a spectrum.

**Figure 13:**
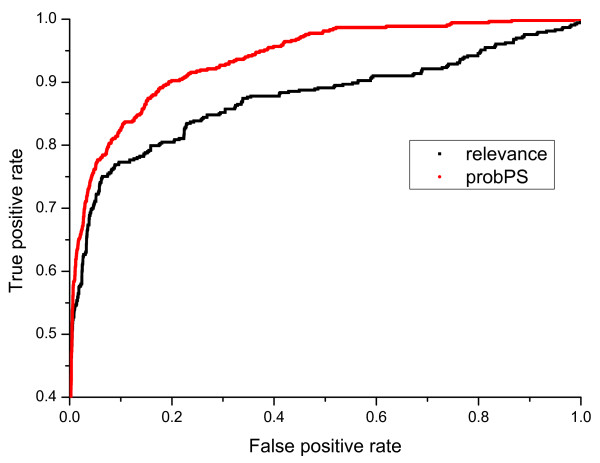
**ROC plots of peak selection performance of AuDeNS and *probPS *acquired from **SwedCAD**data set**.

For the sake of fair comparison, we used the same data sets as AuDeNS, i.e., a training set with 266 LCQ spectra, and a test data set with 20 LCQ spectra. The results of the comparison (shown in Figure 13) suggest that *probPS *outperforms *relevance*. Specifically, when the false positive rate is set to 0.2, *probPS *has a higher true positive rate (0.9) than *relevance *(0.79).

**Figure 14:**
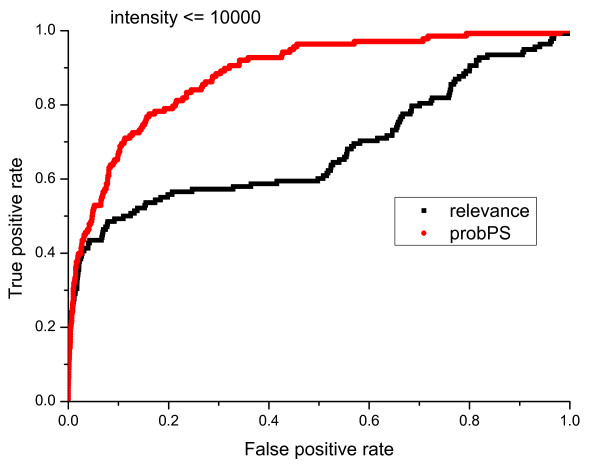
**ROC plots of peak selection performance of AuDeNS and *probPS *for primay peaks with low intensity**.

**Figure 15:**
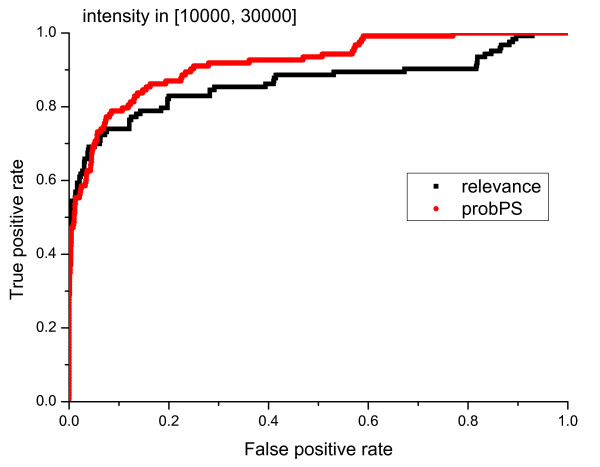
**ROC plots of peak selection performance of AuDeNS and *probPS *for primay peaks with high intensity**.

We also compared the performance of *probPS *and AuDeNS using two categories of primary peaks, high peaks (peak intensity *I >*10000) and low peaks (peak intensity *I <*10000). The results of the comparison are summarized in Figure 14 and 15. It has often been assumed that high intensity peaks are more likely to be valid. However, this is not always true because valid low intensity peaks also exist. For example, ions with small mass/charge ratios, say $b_{2}^{+}$, $y_{1}^{+}$ and $y_{2}^{+}$, are generally of low intensity and can even be invisible in ion trap fragmentation spectra. The results in Figure 6 illustrate that *probPS *is much better than the *relevance *of AuDeNS for selecting low intensity peaks.

### 3.2 Improving *de novo *identification using *probPS*

We investigated whether or not peak selection can improve *de novo *performance. We ran the de novo algorithm [31] in AuDeNS with two types of spectra, one that was generated through peak selection based on *probPS *and the other that was generated through peak selection based on *relevance*. The de novo algorithm in AuDeNS will generate top 30 candidate peptides, and the ranks of correct matched peptides obtained using the two peak selection methods are listed in Table 1. For some spectra, say 03C.1361.1361.2, peak selection using *probPS *is better than that using AuDeNS because *probPS*gave the real peptide a higher rank. For the 01C.0492.0496.2 spectrum, the de novo algorithm failed to identify the correct peptide when peak selection based on *relevance *was used; in contrast, when *probPS *was used, the real peptide was identified. Using *probPS *for peak selection the de novo algorithm correctly identified the top 3 candidate peptides, and overall *probPS *(reporting 8 correct peptides) outperformed *relevance *(reporting 6 correct peptides).

**Table 1:** de novo peptide identification results after peak selection based on *probPS *and *relevance*.

Spectrum	Peptide	Results(relevance)	Results(probPS)
Vacuoles_01C.0492.0496.2	SKAEAESLYQSK	-*^a^*	3rd*^b^*
Vacuoles_01C.0628.0632.2	AADVGADLVGK	1st	1st
Vacuoles_01C.1507.1511.2	TDALDAAGNTTAAIGK	-	-
Vacuoles_01C.1829.1831.2	HGVQELEIELQSQLSK	-	-
Vacuoles_02C.0845.0845.2	AGEFFASAHR	2nd	3rd
Vacuoles_02C.0893.0896.2	NIAVGRPDEATRPDALK	-	-
Vacuoles_02C.1670.1670.2	AAVIGDTIGDPLK	-	-
Vacuoles_03C.0695.0699.2	QYQALGGGANTVAHGYTK	-	-
Vacuoles_03C.1029.1033.2	QYQALGGGANTVAHGYTK	-	-
Vacuoles_03C.1141.1145.2	SLGAAIIYNK	2nd	2nd
Vacuoles_03C.1296.1300.2	LAADTPLLTGQR	2nd	1st
Vacuoles_03C.1361.1361.2	LVDIGTVTAQQAK	11st 3rd	
Vacuoles_03C.1365.1372.2	IRLENEIQTYR	-	IRLEGGEIQTYR
Vacuoles_03C.1437.1441.2	VYVGQGDSGVVYVK	-	-
Vacuoles_03C.1781.1785.2	TLDEQVDQEEFVR	-	-
Vacuoles_03C.1801.1805.2	QISNLQQSISDAEQR	-	-
Vacuoles_03C.1934.1934.2	SLGAAIIFNK	2nd	2nd
Vacuoles_04C.1034.1034.2	NIEQHASDNVNK	2nd	2nd
Vacuoles_04C.2115.2118.2	IGGIGTVPVGR	IGGIGEAPVGR	IGGIGEAPVGR
Vacuoles_04C.3786.3789.2	TAENFANYTGDQGYPGGR	-	-

*^a^*These peptides were not among the top 30 candidate peptides selected by AuDeNS.

*^b^*The correct peptide was ranked 3rd in the top 30 candidate peptides selected by *probPS*.

We performed cross-validation over the 266 LCQ spectra. The 266 spectra were arbitrarily divided into four groups and in each validation round, three groups were used as the training set, and the remaining group was used as the test set. Because some of the spectra were from the same peptide, which might lead to over-fitting, a pre-processing step was performed to ensure that spectra from the same peptide were in the same group. The performance of *probPS *and *AuDeNS *in the four validation rounds are listed in Table 2. The results clearly show the advantage of using *probPS *over *AuDeNS *for peak selection.

**Table 2:** Cross-validation of the performance of *probPS *and *AuDeNS *in improving de novo peptide identification.

			#Correctly identified peptides*^a^*		
			
Round	Methods	#Spectra	Top 1	Top 3	Top 30
1	relevance	61	4	7	14
	*probPS*	61	**4**	**11**	**20**
					
2	relevance	69	7	10	19
	*probPS*	69	**12**	**17**	**26**
					
3	relevance	69	3	8	17
	*probPS*	69	**3**	**10**	**28**
					
4	relevance	67	8	11	23
	*probPS*	67	**11**	**16**	**26**

### 3.3 Identifying tags based on probPS

Ordinary tagging methods directly identify tags on a given mass spectrum. For example, PepNovoTag [36] extracts all substrings of the desired length from the PepNovo reconstruction process, and uses a logistic regression model to evaluate these tags. This strategy suffers from noise peaks in the spectrum. Our method only uses the peaks with high *probPS *values to generate tags. Specifically, our tag identification method (called *probTag*) starts with the top peaks with high *probPS *along with their complementary peaks to find the most reliable neighbor peaks.

We selected the first 1000 spectra reported by SwedCAD as the test data set (spectrum IDs from 1.683.39666.2.dta to 1000.1312.70275.2.dta), and used the remaining spectra in SwedCAD as the training data set. Table 3 summarizes the tag identification performance of *probTag *and PepNovoTag. When the desired tag length was set to 3, *probTag *found 876 of the 1000 tags correctly while PepNovoTag found 790 tags. When the desired tag length was set to 4, *probTag *found 760 correct tags while PepNovoTag found 709 tags. When the desired tag length was set to 5, the two methods found almost the same number of correct tags; however, *probTag *had a higher accuracy (74.67% for *probTag *compared to 61.0% for PepNovoTag).

**Table 3:** Comparison of *probTag *and PepNovoTag (version 3

		Tag Identification Performance		
		
Tag Length	Methods	+	-	Accuracy*^a^*
3	PepNovoTag	790	210	79.00%
	*probTag*	**876**	**106**	**89.21%**
				
4	PepNovoTag	709	291	70.90%
	*probTag*	**760**	**164**	**82.25%**
				
5	PepNovoTag	610	390	61.00%
	*probTag*	**616**	**209**	**74.67%**

*^a ^*Tag identification was "correct" if the tag was contained in the real peptide. Accuracy denotes the ratio of "correct" tag identification.

It should be noticed both PepNovoTag and ProbTag are combinations of peak selection and tagging techniques. This is only an implicit and indirect evidence of the peak selection performance.

## 4 Conclusion and discussion

In this study, we described the dependence between derivate peaks and primary ion intensity in a quantitative manner. The experimental results demonstrate that this quantitative description can help improve the accuracy of peak selection which further improves the performance of de novo sequencing and tag identification.

In addition to the peak attributes used in the study, other attributes like, for example, consecutive ions may prove to further improve peak selection. In general, valid peaks are more likely to have a consecutive ion than invalid peaks. In future work, we aim to incorporate this attribute into our peak selection method.

## Authors' contributions

SH designed and carried out the comparative study, wrote the code, and drafted the manuscript. YW has been responsible for data collection and helped to revise the paper. DB verified and provided discussion on the methodology. HZ brought up the biological problem that prompted the methodological development. SS conceived and developed the peak selection methodology. All authors read and approved the final manuscript.

## Supplementary Material

Additional file 1 **supplementary Figure S1**. The relationship between *r_v_*(*I*) and *I *was also calculated using the data set obtained from an ESI data set provided by Keller. The relationship between *r_v_*(*I*) and *I *shows similar geometric distribution shape, though parameters are not the same due to different experiment conditions. Caption of the Figure: Distribution of *r_v_*(*I*) calculated using the data set from the Keller's Lab. Click here for file
